# Optimizing a global alignment of protein interaction networks

**DOI:** 10.1093/bioinformatics/btt486

**Published:** 2013-09-17

**Authors:** Leonid Chindelevitch, Cheng-Yu Ma, Chung-Shou Liao, Bonnie Berger

**Affiliations:** ^1^Computer Science and Artificial Intelligence Laboratory and ^2^Department of Mathematics, Massachusetts Institute of Technology, Cambridge, MA 02139, USA, ^3^Department of Computer Science and ^4^Department of Industrial Engineering and Engineering Management, National Tsing Hua University, Hsinchu 30013, Taiwan

## Abstract

**Motivation:** The global alignment of protein interaction networks is a widely studied problem. It is an important first step in understanding the relationship between the proteins in different species and identifying functional orthologs. Furthermore, it can provide useful insights into the species’ evolution.

**Results:** We propose a novel algorithm, PISwap, for optimizing global pairwise alignments of protein interaction networks, based on a local optimization heuristic that has previously demonstrated its effectiveness for a variety of other intractable problems. PISwap can begin with different types of network alignment approaches and then iteratively adjust the initial alignments by incorporating network topology information, trading it off for sequence information. In practice, our algorithm efficiently refines other well-studied alignment techniques with almost no additional time cost. We also show the robustness of the algorithm to noise in protein interaction data. In addition, the flexible nature of this algorithm makes it suitable for different applications of network alignment. This algorithm can yield interesting insights into the evolutionary dynamics of related species.

**Availability:** Our software is freely available for non-commercial purposes from our Web site, http://piswap.csail.mit.edu/.

**Contact:** bab@csail.mit.edu or csliao@ie.nthu.edu.tw

**Supplementary information:** Supplementary data are available at *Bioinformatics* online.

## 1 INTRODUCTION

Protein–protein interactions (PPIs) are crucial to a wide variety of cellular processes, and the interacting proteins are likely to evolve with high correlation during the evolution of species (Barabási and Oltvai, 2004; Guo and Hartemink, 2009). Thus, the use of PPI information can help detect functional orthologs, while sequence homology alone is often not sufficient to identify conserved protein complexes (Kelley *et al.*, 2003; Sharan *et al.*, 2005; Zaslavskiy *et al.*, 2009).

Ever since high-throughput experimental screening techniques such as yeast two-hybrid analysis (Formont-Racine *et al.*, 1997; Ito *et al.*, 2001; Uetz *et al.*, 2000), mass spectrometry (Aebersold and Mann, 2003; Bader and Hogue, 2002; Ho *et al.*, 2002) and tandem-affinity purification (Gavin *et al.*, 2002) made protein interaction networks available for several species, efforts have been made in the bioinformatics community to extract useful biological information from these networks. Protein interaction networks provide a more complete and higher-level representation of molecular components than has been available before, and also enable genome-scale understanding of the cell from a systems perspective (Tan and Ideker, 2007). One important goal has been to produce accurate alignments of two or more of these networks, with the expectation that this would help in establishing the biological function of unknown proteins by exhibiting their correspondence with the proteins of another species with known biological function and providing insight into evolutionary dynamics.

Alignments of protein interaction networks can be broadly classified into two categories: *local* and *global* alignments. The distinction is similar to the one made for sequence alignment algorithms. More specifically, local network alignment is concerned with identifying a subnetwork of one species closely matching a subnetwork of another species or having a certain topology (Sharan *et al.*, 2005). Typically, multiple closely matching subnetworks are identified by such algorithms, which may be mutually inconsistent (Singh *et al.*, 2008). On the other hand, global network alignment attempts to map two or more networks as a whole, and their output is a single mapping between the vertices of the networks (Singh *et al.*, 2008). Furthermore, the objective of global alignment of PPI networks is to search for the best consistent mapping between all vertices across the networks, which can reveal evolutionarily conserved functions at the system level. In contrast to local network alignment, relatively little attention has been paid to global network alignment. In the present article, which deals with the global alignment problem, we view this mapping as a bipartite matching, where the vertices on one side of the bipartite graph are the proteins in one network, and the vertices on the other side are the proteins in the other network.

Following the rapidly increasing availability of large PPI networks, many analytical and algorithmic approaches have been developed for their comparative analysis. Previous work on the problem of PPI network alignment includes NetworkBLAST-M (Kalaev *et al.*, 2008), Graemlin 2.0 (Flannick *et al.*, 2009), IsoRank (Singh *et al.*, 2008), IsoRankN (Liao *et al.*, 2009), PATH (Zaslavskiy *et al.*, 2009) and GRAAL (Kuchaiev *et al.*, 2010), although a number of other techniques exist as well (Aladağ and Erten, 2013; Berg and Lässig, 2006; Dutkowski and Tiuryn, 2007; Guo and Hartemink, 2009; Kelley *et al.*, 2003, 2004; Koyutürk *et al.*, 2006; Patro and Kingsford, 2012; Sharan *et al.*, 2005; Srinivasan *et al.*, 2006). A couple of these (Aladağ and Erten, 2013; Patro and Kingsford, 2012) build on a preliminary version of our work (Chindelevitch *et al.*, 2010). Moreover, some of these methods can handle more than two networks.

NetworkBLAST-M uses a new data representation of networks and computes a local alignment by greedily finding regions of high local conservation based on inferred phylogeny. Graemlin 2.0 provides a network aligner to compute both local and global alignments by training a network inference algorithm on a known set of alignments, and then optimizing the learned objective function on the set of all networks. IsoRank first finds pairwise alignment scores across all pairs of networks, obtained by a spectral method on a product graph; it then uses these scores in a greedy algorithm to produce the final global alignment. The more recent IsoRankN uses a different method of spectral clustering on the multiple alignment graph to compute a global alignment of multiple PPI networks. PATH formulates the pairwise network alignment as a graph matching problem and solves its convex and concave relaxations by iteratively updating the weights and following the path of solutions thus created. GRAAL, in contrast, computes a sequence-free pairwise alignment by using the notion of graphlet degree signatures.

As pointed out in the literature (Liao *et al.*, 2009; Zaslavskiy *et al.*, 2009), one of the main difficulties faced by network alignment algorithms is the lack of an accurate and reliable gold standard for evaluation purposes. Another challenge is the computational complexity of network alignment algorithms. The problem of network alignment is a generalization of the intractable subgraph isomorphism problem. A biological challenge is that a network alignment algorithm must efficiently and effectively identify biologically conserved functions.

### 1.1 Contribution

We propose a novel tool, PISwap, based on local optimization, for computing pairwise global alignment of protein interaction networks. The algorithm begins by identifying an optimal global alignment based purely on sequence data, which correctly determines functionally orthologous proteins in many, but not all, cases. To adjust this initial alignment, PISwap uses the intuition that biologically conserved interactions can compensate for mapping proteins whose sequences are not particularly similar to one another. In this way, the topology of the networks is taken into account, and information is propagated from each vertex to its neighbors.

Using the protein interaction networks available for five species, namely, yeast, fly, worm, human and mouse, we pairwise align the first three networks, as well as the human and mouse networks. The results demonstrate the usefulness of the local search technique as well as the functional effectiveness of topologically optimizing the global alignment. Furthermore, we suggest that PISwap can topologically refine other global alignment algorithms at almost no additional cost. More precisely, the local search technique can efficiently fine-tune other approaches when used as a postprocessing step. We also test PISwap on pairs of yeast and fly networks, as well as their *randomized* versions. The results also demonstrate the algorithm’s robustness to noise in network data. Finally, we explain how specific information produced by PISwap can produce insights into the evolutionary dynamics of protein interactions.

## 2 METHODS

### 2.1 Problem formulation

We consider the global alignment of a pair of protein–protein interaction (PPI) networks. Each network is represented by a graph whose vertices correspond to proteins, and there is an undirected edge between two vertices if and only if the corresponding proteins interact. Given a pair of PPI networks and a list of pairwise sequence similarities between proteins in the two networks computed according to some criterion, global alignment aims to find an optimal mapping between the proteins of the two networks that best represents conserved biological function. We formulate network alignment as a graph-theoretic problem as follows.

Consider two PPI networks 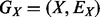 and 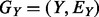 whose edges represent protein interactions. Let 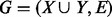 be an edge-weighted complete bipartite graph consisting of the vertex subsets *X* and *Y*, with each edge 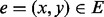 associated with a non-negative edge weight *s*(*e*), which represents the sequence similarity between vertices 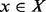 and 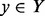, bounded by the length of protein sequences. That is, 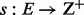 denotes a non-negative integer *sequence similarity* function on the edges of *G*, where the sequence similarity of a pair of proteins could be, for instance, the BLAST Bit-value of the sequences as retrieved from Ensembl (Hubbard *et al.*, 2009).

A mapping 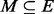 of *G* is defined to be a subset of edges such that no two edges in *M* share an endpoint. In addition, given a mapping *M*, we define a non-negative integer *topology similarity* function 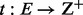. For an edge, 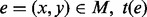 represents the topology similarity between the neighborhoods of 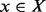 and 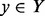, i.e. the number of edges in these neighborhoods conserved by the mapping *M*. To be more precise, for each edge, 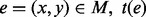 is the number of edges between the neighborhoods of *x* and 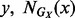 and 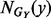, respectively, which are also in the mapping *M*, i.e. 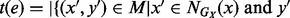 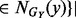. Our objective is to find a mapping *M* such that the following weight function *w* is maximized:
(1)
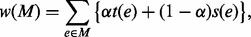

where 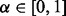 is a parameter that controls the importance of the network topology similarity relative to sequence similarity.

The above weight function is a convex combination of two terms: the topology similarity function *t* and the sequence similarity function *s*. Tuning the parameter α allows us to change the relative importance of PPI network data in finding the optimal global alignment. At one extreme, 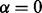 implies that no network data are used, whereas at the other extreme, 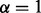 indicates that only network data are used. This formulation of the problem is known to be NP-hard in general, not only to solve, but also to approximate (Sahni and Gonzales, 1976), which means that it does not admit a polynomial-time algorithm unless P = NP.

Because the size of the search space grows exponentially with the number of proteins in each network, we use a local search technique adapted from other NP-hard optimization problems, which is described in detail in the following subsection.

### 2.2 Algorithm

Here, we simply explain the basic idea of our method, leaving its analysis to the Appendix (Supplementary Information). The main purpose of the algorithm is to refine any global alignment of pairwise PPI networks via local optimization techniques. Based on conserved functional properties within PPI networks, our algorithm can fine-tune arbitrary pairwise global alignments. The key concept of our method is to apply a local search heuristic, which is widely used in the combinatorial optimization field, to iteratively improve the initial mapping while taking into account both the sequence score and the topology score of the mapping.

From a variety of local search methods, we make use of the idea of the 2-Opt algorithm, which was first proposed by Croes (1958), and also generalize to the 3-Opt algorithm. The 2-Opt algorithm is one of the most famous heuristics for the well-known *Traveling Salesman Problem*, (Lawler *et al.*, 1985). Given a set of cities, the Traveling Salesman Problem (TSP) is to find an ordering of cities that minimizes the total length of the tour when visiting all the cities in some order and returning to the starting city. The basic concept of the 2-Opt algorithm is simple. A move deletes two edges of the original tour, thus breaking the tour into two paths, and then reconnects those paths by swapping these edges.

Furthermore, 2-Opt outperformed almost all the local search and greedy algorithms in experimental results for TSP (Johnson and McGeoch, 1997). More precisely, 2-Opt (or *k*-Opt) gave better final tours than other local search algorithms for TSPLIB instances (Johnson and McGeoch, 1997) with respect to both approximation ratio and running time. The 3-Opt technique considers three, rather than two, edges of a mapping in each round and determines whether the objective function can increase by swapping these edges. The details of the 3-Opt technique are presented in the Appendix (Supplementary Information).

We use a local search approach to the pairwise PPI network alignment problem in a manner similar to 2-Opt as follows: when given a maximum weighted bipartite mapping 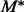 in 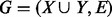, we define a vertex subset 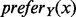 for each 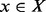, which consists of the *c* highest-weighted neighbors of *x* in *Y* (where the weight of a neighbor of *x* is given by its sequence similarity to *x*). Similarly, for every 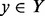, a vertex subset 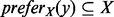 is defined to consist of the *c* highest-weighted neighbors of *y* in *X*. The *c* is some relatively small constant chosen ahead of time. It can be shown (Johnson and McGeoch, 1997) that *c* = 20 suffices for most practical applications. Our aim is to repeatedly find a candidate 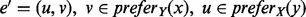 to swap with 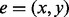, where 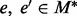, such that the weight of the new mapping, 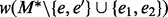, where 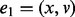 and 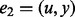 are the edges obtained by swapping *e* and 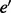, is higher than 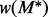.

## 3 RESULTS

In this study, we began by comparing the performance differences between 2-Opt and 3-Opt when using PISwap to refine the mappings obtained by the well-known *Hungarian algorithm* (Kuhn, 1955), which only depends on sequence similarity. Next, we examine the performance of PISwap on initial mappings produced by other popular global alignment algorithms, GRAAL (Kuchaiev *et al.*, 2010), IsoRank (Singh *et al.*, 2008) and PATH (Zaslavskiy *et al.*, 2009). Finally, we verify the robustness of PISwap to noise in PPI data. We do so by testing PISwap on the yeast and fly networks as well as their *randomized* versions to refine the initial mappings derived from the three global alignment tools above.

The key concepts of the above global alignment algorithms are quite different. GRAAL is a sequence-free global network alignment tool. Every vertex in a PPI network is associated with a 73-component graphlet degree vector, which counts the number of different graphlets that the vertex touches; this vector can be considered a *signature* of the vertex. GRAAL uses a greedy algorithm to find a pairwise alignment maximizing the total similarity of the vectors corresponding to the pairs of matched vertices in two PPI networks. On the other hand, IsoRank uses spectral graph theory to compute an alignment score for each pair of vertices in two PPI networks. It considers two vertices to be a good match if their respective neighbors are also good matches. Hence, the score of a pair depends on the score of its neighbors, which in turn depends on the score of its neighbors, and so on. IsoRank combines topology scores and sequence-based BLAST bit scores and extracts a matching in a greedy manner. Like IsoRank, the PATH algorithm also combines the number of conserved interactions with a sequence similarity score. Based on the techniques of concave and convex relaxations, it aligns two PPI networks by solving a convex relaxation in the beginning. Next, a linear combination of the convex and concave relaxations is solved iteratively by increasing the weight of the concave relaxation and following the path of feasible solutions thus created. The algorithm stops when the solution reaches a corner of the set of doubly stochastic matrices.

We selected the following five eukaryotic species for our experimental analysis: *Caenorhabditis elegans* (worm), *Drosophila melanogaster* (fly), *Saccharomyces cerevisiae* (yeast), *Homo sapiens* (human) and *Mus musculus* (mouse), each of which has complete PPIs available. For convenience, we abbreviated each of the five species as follows: CE = *C.elegans*, DM = *D.melanogaster*, SC = *S.cerevisiae*, HS = *H.sapiens* and MM = *M.musculus*. We tested PISwap on four pairs: DM versus SC, CE versus SC, CE versus DM and HS versus MM. Note that, because human and mouse are at a substantial evolutionary distance from SC, CE and DM, we did not perform a comparison between them and the other three species.

Recall that the goal of PISwap is to topologically refine a global network alignment while maintaining its functional consistency. The topological information in PPI networks can help identify conserved functions that protein sequence homology alone cannot easily detect. In biological systems, the function of a protein depends on its 3D structure, which is usually determined by its protein sequence. However, in some cases, proteins with similar functions and 3D structures might have different sequences. For instance, a circular permutation is the rearrangement of a protein sequence. The *N*-terminal and *C*-terminal regions of a protein might be interchanged while their 3D folding structures remain the same. The rearranged sequences cannot be easily detected by sequence alignment. Several natural examples of circular permutation have been reported in the literature (Lindqvist and Schneider, 1997), such as bacterial β-glucanases, swaposins, glucosyltransferases, β-glucosidases, etc. Thus, the functions of these proteins cannot be annotated by sequence alignment alone. On the other hand, topological properties of a PPI network could be helpful for understanding the possible functions of these proteins (Komili *et al.*, 2007; Kuchaiev *et al.*, 2010; Memišević *et al.*, 2010).

For these reasons, we examine the similarity of topological neighborhoods and functional consistency for evaluating a global network alignment. For the former, we use the edge correctness (EC) ratio (Kuchaiev and Pržulj, 2010), but modify the definition’s normalization because the size of the PPI networks varies considerably in this study. The EC ratio is defined as follows:
(2)
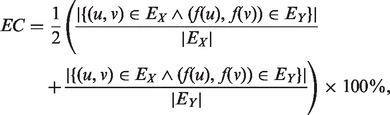

where *E_X_* and *E_Y_* are the edge sets of *G_X_* and *G_Y_*, respectively, and *f*(*u*) and *f*(*v*) are the vertices in *G_Y_* that are aligned with *u* and *v*, respectively, in *G_X_* via a network alignment *f*. A higher EC ratio means that the alignment between two organisms preserves more interactions.

We also use the functional coherence (FC) value to measure the functional consistency of the mapped proteins following the method proposed by Singh *et al.* (2008). The FC value of a mapping is computed as the average pairwise FC of the protein pairs that are aligned. A higher FC score indicates that the proteins in the mapping perform more similar functions. The method for computing FC values can be summarized as follows. First, the Gene Ontology (GO) terms corresponding to each protein are collected. GO terms are a hierarchical description of protein functions. Then, each GO term is mapped to a subset of the so-called standardized GO terms, which in this case are its ancestors lying within a distance five from the root of the GO tree. Finally, the similarity between each pair of aligned proteins is computed as the median of the fractional overlaps of their corresponding sets of standardized GO terms. The FC of each protein pair is defined as:
(3)
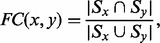

where *S_x_* and *S_y_* are the GO term sets of protein *x* and protein *y*, respectively, for 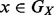 and 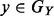. Note that only 60–70% of the proteins in any of the aligned networks have an annotated GO ID, comparable with the fraction of all known proteins included in GO.

### 3.1 Implementation

All the PPI networks we used were constructed by combining the data from BioGRID (Breitkreutz *et al.*, 2008), Database of Interacting Proteins (DIP; Salwinski *et al.*, 2004), Human Protein Reference Database (HPRD; Keshava Prasad *et al.*, 2009), and retrieved from the Isobase Web site (Park *et al.*, 2011). In total, these five PPI networks contained 87 737 proteins and 114 897 known interactions. The number of vertices (edges) in each PPI network is 19 756 (5853), 14 098 (26 726), 6659 (38 109), 22 369 (43 757) and 24 855 (452) for CE, DM, SC, HS and MM, respectively. We take into account both raw and normalized sequence data, retrieved from the Isobase Web site as well. The raw BLAST scores used are computed as 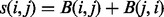, where 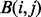 is the value given by BLAST on input *i* and *j* (this is because BLAST sometimes produces asymmetric results). The normalized scores were computed as 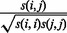 (Singh *et al.*, 2008), and resulted in values between 0 and 1. For PISwap, the ratio of α to 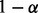 can be thought of giving the relative weight of sequence information to topology information. We found that choosing α to make this relative weight half of what it is for the initial mapping was a good rule of thumb. In other words, as the initial mapping was based purely on sequence information, we chose α to favor the topology twice as much as the initial mapping did, in the first reported experiment. More precisely, we used the equation 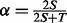 to adjust the α value in our experiments to achieve the best setting, where 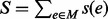 is the total sequence similarity score and 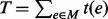 is the total topology similarity score. Hence, the equation determines the α value depending on the fraction of sequence score and topology score of an initial mapping. In the other experiments without sequence information, the α value is set to 1.

In addition to performing our experiments on the five PPI networks based on the 2010 releases of the underlying databases, we also performed all of our experiments on the less recent 2008 releases, using the networks at Isobase (Park *et al.*, 2011). We provide the detailed results of these experiments in the Supplementary Materials. Because the new networks remain similar to their earlier versions, these results are qualitatively very similar to the ones reported in the main text. The FC values changed by <0.02 in all of the experiments, with the exception of the alignments of the CE and DM networks by GRAAL and PATH, where it increased by 0.024 and 0.027, respectively, as well as PISwap’s refinement of their alignment by PATH, where it increased by 0.025. The EC ratios did not change by >2% in either direction, except in the case of the refinement of IsoRank’s network alignment, where they increased by 2.2 (for the CE–DM alignment) to 3% (for the HS–MM alignment). The numbers of edge swaps required in each case remained within a factor of 2 across the experiments, with the exception of the HS–MM network alignment, where they decreased from 476 to 185 during the refinement phase. Interestingly, the fractional improvement of IsoRank with PISwap decreased substantially, from an average of 35% in the more recent network pairs to 19% in the less recent network pairs. The running times required to perform the alignments and the refinements frequently increased, sometimes by as much as 100%, whereas a handful of experiments ran faster by at most 25%.

The algorithm was implemented in Python 2.6 using the NetworkX (Hagberg *et al.*, 2008) package, as well as Joris van Rantwijk’s implementation of the maximum-weight mapping algorithm based on the blossom method for finding augmenting paths and the primal–dual method for finding a maximum-weight matching (Galil, 1986). All experiments were performed on a desktop with a 64-bit architecture running Windows 7 with an Intel Core i7-2600 CPU and 16-GB of RAM.

### 3.2 Performance of 2-Opt and 3-Opt

We first compared 2-Opt with 3-Opt when using PISwap on the initial topology-free mappings produced by *Hungarian algorithm*. The result of the first experiment is summarized as follows. PISwap improves the EC ratio in each pair while at the same time maintaining consistency of their FC values. This shows that our algorithm is performing its goal of achieving a higher topology similarity while retaining a consistent sequence similarity. Because 3-Opt considers more candidate edges for swapping than 2-Opt, 3-Opt results in higher EC ratios and compares favorably with 2-Opt.

Table 1 illustrates the results of 2-Opt and 3-Opt when using the output of the *Hungarian algorithm* as the initial mapping with the raw sequence similarity data (the results with normalized similarity data are qualitatively very similar). The number of swaps required is 81 (49) for the mapping between DM and SC, 41 (66) for that between CE and SC, 53 (173) for that between CE and DM and 26 (31) for that between HS and MM. In each pair, the first number is the number of swaps made by 2-Opt and the second, by 3-Opt. Note that these are significantly higher than the diameter (length of the path between the two most distant nodes) of the PPI networks, which are 14, 11, 9, 14 and 10 for CE, DM, SC, HS and MM, respectively, meaning that information may have had a chance to propagate to all of the vertices.

**Table 1.** Evaluation of alignments based on the initial mappings produced by *Hungarian algorithm*

	DM–SC			CE–SC			CE–DM			HS–MM		
	Initial	2-Opt	3-Opt	Initial	2-Opt	3-Opt	Initial	2-Opt	3-Opt	Initial	2-Opt	3-Opt
Number of swaps	0	81	49	0	41	66	0	53	173	0	26	31
EC ratio	0.49%	0.67%	0.68%	0.69%	1.02%	1.16%	0.48%	0.91%	1.39%	24.66%	31.97%	32.28%
Functional coherence	0.596	0.595	0.593	0.294	0.294	0.294	0.395	0.394	0.393	0.46	0.469	0.469
Running time (seconds)	264	6	15	296	4	20	1791	16	86	28 145	97	917

*Note*: CE: *C.elegans*; DM: *D.melanogaster*; SC: *S.cerevisiae*; HS: *H.sapiens*; MM: *M.musculus.*

The resulting EC ratios clearly show substantial improvement in each pair; meanwhile, there are only slight differences between the FC values of the initial mappings and of those refined by PISwap (for both 2-Opt and 3-Opt). We note that the EC ratio is affected by the number of edges in the networks by its definition. This is the reason why the EC ratio of HS–MM is larger than those of the other pairs, as the mouse PPI network only has 452 edges.

Moreover, despite the analysis presented in the Appendix (Supplementary Information), the running-time of our algorithm is actually dominated by the preprocessing step, that of finding a maximum weight bipartite matching. This is because the running time is cubic in the size of the input, whereas the number of iterations in the main loop of the algorithm is typically <2000 even when we use 3-Opt. Thus, the upper bound presented in the analysis is overly pessimistic. We use the value 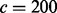 for our experiments, as this is close to the maximum degree Δ of the input networks (which is 184, 190, 323, 243 and 11 for *C.elegans*, *D.melanogaster*, *S.cerevisiae*, *H.sapiens* and *M.musculus*, respectively).

Based on the above discussion, the 3-Opt technique performs a better refinement of global alignments of protein interaction networks, with only minimal time cost relative to the expensive initial alignment step. In the rest of this article, we focus on the performance of 3-Opt PISwap when using initial mappings produced by different types of global alignment algorithms, such as sequence-free alignment and integrated approaches, which combine sequence data with topology information.

### 3.3 Refining GRAAL, IsoRank and PATH

In this experiment, we apply the 3-Opt PISwap to refine the initial mappings derived from GRAAL, IsoRank and PATH, respectively. As mentioned above, GRAAL is a sequence-free network alignment algorithm based purely on topology information, whereas IsoRank and PATH integrate sequence data with network information to produce their mappings.

The three alignments are computed using the default settings. For GRAAL, the α value, which determines the contribution of the graphlet signature of each vertex, is set to 0.8. Because GRAAL begins with an initial pair of vertices randomly, we run each test 30 times and average the results over the 30 runs. In addition, we set the IsoRank parameter α, controlling the weight of topology similarity relative to sequence similarity, to 0.6. The maximum number of iterations, *K*, for the Power Method procedure of IsoRank, is set to 3. On the other hand, the λ value, similar to α in IsoRank and PISwap, is set automatically in PATH. The constraints on the largest steps allowed and the minimal increment in λ are set to 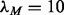 and 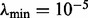 for the iterative relaxation in PATH.

**Fig. 1.**
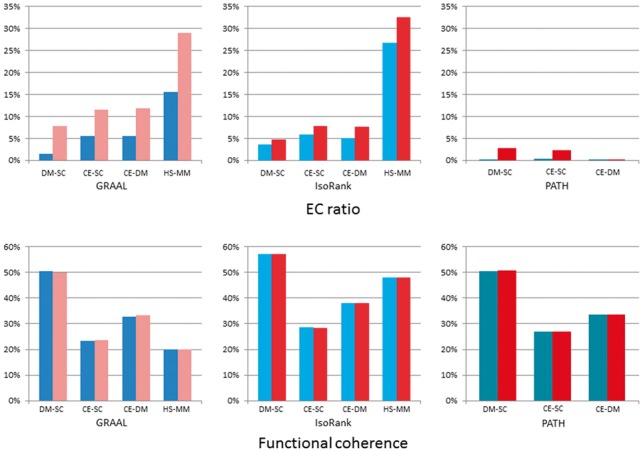
Evaluation of the refinement of the initial mappings obtained by GRAAL, IsoRank and PATH; each of the blue-series and red-series bars, respectively, represents the result before and after refinement by PISwap

Figure 1 shows the performance of PISwap on refining the initial mappings obtained by GRAAL, IsoRank and PATH. Note that the results are produced with normalized sequence data because IsoRank suggests that normalized BLAST scores be used. The increase in the EC ratio for each pair of species in Figure 1 represents a significant improvement over GRAAL, IsoRank and PATH. In particular, the results demonstrate that the 3-Opt PISwap can even identify more conserved interactions than were preserved by the initial mapping from GRAAL, which is based purely on network information. We remark that the HS–MM alignment for PATH is omitted because the program runs for too long.

The numbers of swaps required were 1351, 191 and 650 for the mapping between DM and SC, 453, 240 and 162 for that between CE and SC, 473, 156 and 25 for that between CE and DM, and 71, 476 for that between HS and MM. In each pair, the first number corresponds to GRAAL, the second, to IsoRank and the third, to PATH; there are only two numbers for the HS–MM alignment because PATH exceeded the time limit on this pair of networks. (The numbers of pairs produced by the three alignment tools were 4928, 4254 and 4928 for DM–SC; 2745, 3691 and 2745 for CE–SC; 2745, 7508 and 2745 for CE–DM; and 218, 18 387 for HS–MM. Note that GRAAL and PATH map all the nodes in the smaller network, whereas IsoRank may leave some nodes unmapped because these nodes may not be able to make a non-zero contribution to the objective function guiding the alignment). Although the results show refinement on all initial mappings, the refining effects on the three alignment tools are different. Because GRAAL is a sequence-free global alignment algorithm, we select 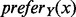 for each vertex 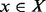 based on the topology similarity between neighbors of *x* in *Y* and *x* instead (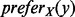 for each vertex 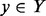 is defined similarly). For every pair, the refined EC ratios are almost twice as large as those of the initial mappings obtained by GRAAL (1.42% versus 7.85% for DM and SC, 5.57% versus 11.57% for CE and SC, 5.47% versus 11.79% for CE and DM and 15.53% versus 28.92% for HS and MM). In contrast, the EC ratios of the refined mappings, originally derived by IsoRank, increase relatively modestly. The results show at least a 20% improvement on the EC ratios (32.8% for DM–SC, 31.5% for CE–SC, 52.4% for CE–DM and 21.6% for HS–MM). On the other hand, compared with GRAAL and IsoRank, the initial mappings obtained by PATH have small EC ratios. Figure 1 demonstrates a substantial improvement over the EC ratios in PATH. For each pair, the refined EC ratio is at least five times as large as the original one (0.25% versus 2.72% for DM–SC, 0.41% versus 2.22% for CE–SC and 0.05% versus 0.25% for CE–DM). This suggests that the performance of PISwap may differ based on the properties of the initial mapping. We defer the discussion of the possible reasons for these differences to Section 4.

Similar to the previous experiment, the FC values remain stable with only about 1% change for each pair when we refine GRAAL, IsoRank and PATH (except in the case of CE–DM derived by GRAAL, where we see a 2% change). For example, the FC values for the pairwise alignment of yeast and fly produced by GRAAL, IsoRank and PATH were 0.506, 0.572 and 0.505, respectively. After our swap operations, the FC values of the refined mappings were 0.499, 0.571 and 0.505, respectively, compared with the original ones. In other words, the 3-Opt PISwap retains the functional consistency of the initial mappings.

In addition, the running time of the entire procedure of PISwap is dominated by the initial alignment algorithms as well. For example, GRAAL, IsoRank and PATH spend ∼77, 57 and 513 minutes, respectively, to obtain the initial mappings in the DM–SC case. On the other hand, PISwap takes only 4, 0.5 and 1.8 min, respectively, for the three algorithms.

### 3.4 Robustness

One of the challenges for protein network alignment is that known PPI networks are both incomplete and inaccurate (Liao *et al.*, 2009). The technical false-positive errors arise from limitations of the experimental procedures, such as yeast two-hybrid analysis (Han *et al.*, 2005). Moreover, a significant percentage (nearly 20%) of interactions observed by the two-hybrid method might not be biologically relevant (Barabási and Oltvai, 2004). Thus, it is critical to measure the fault tolerance of network alignment algorithms.

To verify the robustness of PISwap to noisy PPI data, we evaluate the performance of our algorithm when it aligns PPI networks with their *randomized* versions. More precisely, we test PISwap on pairs of yeast, fly and their *randomized* versions. In this experiment, we evaluate robustness based on how much similarity and consistency the swap operations preserve compared with the original PPI network alignments. We refer to (Kuchaiev *et al.*, 2010; Przulj *et al.*, 2004) and select the *geometric random graph* model to generate the *randomized* networks. Other random network models, such as scale-free networks, which preserve the power-law degree distribution of the PPI networks, might not represent the data appropriately in this experiment because they are strongly constrained in their structures; such constraints might transfer topology similarity to the models for yeast and fly. On the other hand, the PPI networks are known to be well represented by the geometric random graph model (Kuchaiev *et al.*, 2010; Przulj *et al.*, 2004). The intuition behind this model is based on the observation that proteins interact with other proteins in some biochemical space, which implies that proteins closer together in this space are more likely to have an interaction. This theoretical model, in which proteins are represented by vertices in a metric space and are connected by an edge if they lie within a specified distance of each other, requires only a few tunable parameters.

For the PPI networks of yeast and fly, we used the geometric random graph model (Higham *et al.*, 2008) by applying the igraph package (Csardi and Nepusz, 2006) to generate 10 *randomized* networks, each of which contains the same number of vertices and edges as SC and DM, respectively. We aligned each random network with a real PPI network by using GRAAL and IsoRank—more precisely, a randomized network for DM versus the real PPI network for SC (DM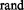-SC) and the real PPI network for DM versus a randomized network for SC (DM-SC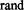). Then, we performed 3-Opt PISwap on these initial mappings obtained by the two alignment tools to evaluate the performance of our algorithm. Because a *randomized* network does not have sequence information, we selected 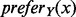 and 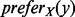 for each vertex based on topology similarity instead. In this experiment, we ran every alignment tool on each pair 10 times and averaged results over the 10 runs. We note that the experimental results for PATH were skipped because the program ran for too long without sequence similarity scores.

**Fig. 2.**
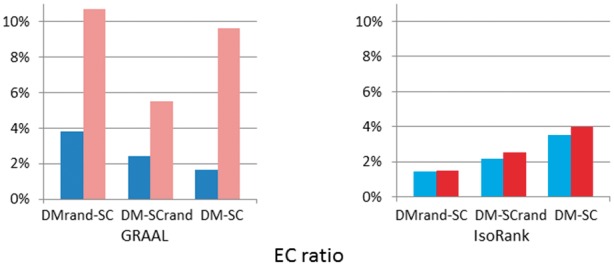
Simulation experiments for robustness of PISwap; each of the blue-series and red-series bars, respectively, represents the result before and after refinement by PISwap

Figure 2 illustrates the performance of PISwap. The results of this experiment show that our algorithm can still consistently improve the EC ratio even if one of the two aligned networks is randomized. The numbers of swaps required were 1508 (32) for the mapping between 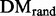 and SC and 1057 (164) for that between DM and 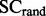. In each pair, the first number corresponds to GRAAL, and the second, to IsoRank. In the case of GRAAL, the EC ratios refined by PISwap are at least twice as large as those of the initial mappings (3.82% versus 10.71% for 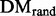-SC and 2.43% versus 5.49% for 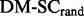). Compared with the refinement of the pairwise alignment of real PPI networks (the number of swaps required is 1960 and the improvement on EC ratio is 1.65% versus 9.6% for DM-SC), the refining effect is acceptable. On the other hand, the EC ratios of the refined mappings that were originally derived by IsoRank can still gain at least a 4% improvement (4.2% for 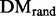-SC and 18.6% for 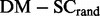), compared with the 12.7% improvement for DM-SC. This suggests that the refining effects are similar to those of the real PPI network alignment between DM and SC. Thus, these simulation experiments suggest that our algorithm is robust to errors in PPI data.

## 4 DISCUSSION

### 4.1 PISwap

We have presented an algorithm, PISwap, for refining an arbitrary global alignment of protein interaction networks while maintaining its FC. In PISwap, the parameter α plays an important role because it determines the relative importance of the topology data and the sequence data. Although our objective function is identical to that used in the IsoRank algorithm (Singh *et al.*, 2008), there are a number of important differences. IsoRank performs a random walk on the graph 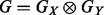, the tensor product of the two networks, where at each step, the walk is restarted with probability 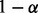 at a node 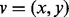 in *G* chosen at random from the distribution proportional to the sequence similarity 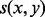. On the other hand, PISwap can be thought of as performing a walk on the set of all matchings in the complete bipartite graph, and this walk is not random, but has the property that every step increases the value of the objective function. Another difference from IsoRank is that the output of IsoRank in terms of the pairwise alignment scores *R_ij_* changes continuously with α, whereas in PISwap, the set of possible matchings is discrete and the interval [0,1] can be subdivided into non-overlapping subintervals such that on each one, the resulting matching is the same. Finally, PISwap is not based on a spectral method unlike both IsoRank and IsoRankN.

### 4.2 Detailed settings

In this study, we intend for PISwap to be used as a *booster* to any kind of initial alignment program and demonstrate that the tool can effectively refine mappings obtained by state-of-the-art alignment algorithms; however, the refining effects on these initial mappings are different. The major cause of the difference is the nature of these network alignment tools whose algorithmic principles mean that PISwap begins with different quality mappings. Moreover, these starting solutions may be crucial for our local search technique even though 3-Opt is powerful enough to vastly improve on them. Note that Table 1 shows that 3-Opt is much better than 2-Opt; however, *k*-Opt, 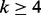, is not sufficiently better than 3-Opt to justify the additional running time. This result is analogous to the scenario of TSP.

Another reason for a distinct improvement over these initial mappings is the selection of 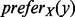 and 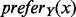, which determine the candidates to be swapped, i.e. the search space. Better candidate neighborhoods for each vertex lead to better local optima. For example, we tested PISwap on the mappings derived by IsoRank by using both sequence and topological similarity to select the candidates for swap operations. Our results show that the candidate neighborhood selected by sequence similarity produces better performance.

For the evaluation of FC, the FC value depends on GO terms, many of which are annotated by sequence alignment. Thus, the refinement of PISwap can be thought of as a topological improvement, which can compensate for a sequence-based alignment and discover functional orthologs that are not derived by sequence-only approaches. This would be the reason that the FC values sometimes decrease slightly after the mappings are refined by our algorithm. We note that the FC value that GRAAL gets for aligning the human and mouse networks is low because the number of matched proteins in the mapping obtained by GRAAL is only 290, and most of these are not annotated by GO terms.

### 4.3 Evolutionary model

Finally, although the edge-swapping technique was originally inspired by the field of combinatorial optimization, one can speculate that it can actually give us insights into the way two networks evolved from a common ancestor. If the networks belong to two closely related species, it is conceivable that at the outset, the proteins of the two networks were essentially identical in sequence, and hence, their correspondence could be determined exclusively on the basis of sequence information. Suppose, however, that as the two species evolve, a pair of proteins in one of them have traded functions relative with one another. In that case, reconstructing the initial correspondence would require precisely an edge swap. Hence, the number of edge swaps required to recover the biologically ‘correct’ mapping from the initial matching based purely on sequence information could possibly tell us about the number of such evolutionary events that have taken place since the initial divergence of the species.

Comparing the network alignment problem with the (simpler) sequence alignment problem, one could say that edge swaps at the network level are the analog of compensatory mutations at the sequence level. One could then argue that, just as compensatory mutations can provide important clues for the evolutionary history of the sequences, function exchanges (represented by edge swaps) can provide important indications for the evolutionary history of the protein interaction networks. Unfortunately, function exchanges are much more difficult to detect than compensatory mutations, as network data are noisy, incomplete and unreliable (Singh *et al.*, 2008). Nevertheless, an algorithm such as PISwap could be adapted to estimating the number of function exchange events that have taken place during the evolutionary process.

Although evolutionary events other than exchanges of function, such as duplications, insertions and deletions of proteins, have certainly taken place (Koyutürk *et al.*, 2006), this approach can still yield useful knowledge. In addition, the evolutionary distance between two species could in principle be computed from the number of evolutionary events (including function exchanges) that have taken place, and could perhaps provide a more accurate estimate than the (appropriately defined and weighted) edit distance between two orthologous sequences present in those two species, as it would in some sense encompass all the protein sequences at once.

Recently, a considerable amount of research explored system approaches for computing evolutionary distances between organisms by using metabolic pathway information. In particular, several studies investigated the topology of metabolic networks between organisms to speculate on their phylogenetic relationships. Zhang *et al.* (2006) compared the topological properties of metabolic pathways to define an evolutionary distance between organisms. Mano *et al.* (2010) considered the topology of pathways as chains and used a pathway alignment method to classify species. Furthermore, Kuchaiev *et al.* (2010) defined a distance metric between two species by using the EC ratio of pairwise metabolic network alignments and reconstructed phylogenetic trees. Global alignment of biological networks may reveal the evolutionary relationship from a systems-level perspective (Ma *et al.*, 2013). It would be of great interest to have a better understanding of phylogeny by using our global alignment algorithm on biological networks.

## Supplementary Material

Supplementary Data

Supplementary Data
