# Supplementary material for: Optimizing a global alignment of protein interaction networks
Source: Bioinformatics. 2013 Sep 17;29(21):2765–73. doi: 10.1093/bioinformatics/btt486 (PMC3799479; doi:10.1093/bioinformatics/btt486)
Supplement: Supplementary Data [file supp_29_21_2765__index.html]

Optimizing a Global Alignment of Protein Interaction Networks — Optimizing a global alignment of protein interaction networks — Optimizing a global alignment of protein interaction networks — Supplementary Data 

# Optimizing a global alignment of protein interaction networks

## Supplementary Data

files

**Files in this Data Supplement:**

- Supplementary Data - pdf file
- Supplementary Data - pdf file
