# Supplementary material for: Optimizing a global alignment of protein interaction networks
Source: Bioinformatics. 2013 Sep 17;29(21):2765–73. doi: 10.1093/bioinformatics/btt486 (PMC3799479; doi:10.1093/bioinformatics/btt486)
Supplement: Supplementary Data [file supp_btt486_piswap_final_revision_0730appendix.pdf]

Supplementary Information

Optimizing a Global Alignment of Protein Interaction Networks

Leonid Chindelevitch, Cheng-Yu Ma, Chung-Shou Liao and Bonnie Berger

This appendix presents a formal description of our algorithm and a running-time bound with proof.

Algorithm

Local search heuristics do not appear to perform well from a theoretical point of view. Papadimitriou and Steiglitz [7] have shown that no local search algorithm (like `2-Opt`) that takes polynomial time per move can guarantee a constant approximation ratio for TSP unless P = NP. In addition, it has been shown that a sequence of exponential moves might be required by `2-Opt` before halting [6] and an analogous result [2] has been extended to `3-Opt` and k-`Opt`.

Although the worst-case analysis of the `2-Opt` algorithm is pessimistic, the average-case analysis is considerably more optimistic. A significant discovery [2] has shown that the expected approximation ratio to the optimum is bounded by a constant. A similarly optimistic result for running time has been obtained as well. That is, the expected number of moves is polynomially bounded. Furthermore, `2-Opt` outperformed almost all the local search and greedy algorithms in experimental results for TSP [3]. More precisely, `2-Opt` (or k-`Opt`) gave better final tours than other local search algorithms for TSPLIB instances [3] with respect to both approximation ratio and running time.

The technique of iterative edge swaps is, however, not limited to TSP. For example, Arya *et al.* [1] proved the approximability of the well-known facility location problem with local search techniques. Another example, Pardalos *et al.* [8] demonstrate that the traveling salseman problem can be easily formulated as a special case of the quadratic assignment problem and the k-opt alorithm also can be apply on it. It is also the basis of an algorithm for graph randomization, which attempts to produce a random graph with a given degree distribution [10]. It can be shown [9] that the corresponding Markov chain converges to the uniform distribution on the set of all connected simple graphs with the given degree distribution, thus giving an exact algorithm.

In this study, without loss of generality, the initial mapping can be considered as the special case of our problem with $\alpha = 0$ (i.e. one where only sequence information is used), which is the problem of finding a maximum-weight mapping in a bipartite graph. In other words, a maximum-weight mapping in the bipartite graph can be obtained by joining pairs of proteins in the two networks, where the weight of an edge is given by the sequence similarity of the two proteins. This mapping can be obtained by the well-known *Hungarian algorithm* in polynomial time [5] and sped up with extensive use of priority queues and decomposition techniques [4]. The initial mapping can also be replaced by an arbitrary global alignment of pairwise PPI networks based on other approaches.

We apply both the `2-Opt` and `3-Opt` techniques in our algorithm. The `3-Opt` technique, compared with `2-Opt`, considers at most three edges of a mapping in each round and determines whether the objective is improved by swapping these edges. Given a maximum weighted bipartite mapping M^* in $G = (X \cup Y, E)$, we define a vertex subset $prefer_Y(x) \subseteq Y$ for each $x \in X$ such that $prefer_Y(x)$ consists of highly weighted neighbors of x in Y and the size of $prefer_Y(x)$ is bounded by a constant c. For every $y \in Y$, a vertex subset $prefer_X(y) \subseteq X$ is similarly defined to consist of highly weighted neighbors of y in X and its size is also bounded by c. Our aim is to repeatedly find candidates $e' = (u, v)$ and $e'' = (p, q)$, v, $q \in prefer_Y(x)$, u, $p \in prefer_X(y)$, to swap $e = (x, y)$ with, where e, e', $e'' \in M^*$, such that the

weight of the new mapping $w(M^* \setminus \{e, e'\} \cup \{e_1, e_2\})$ or $w(M^* \setminus \{e, e', e''\} \cup \{e_1, e_2, e_3\})$ increases, where $e_1 = (x, v)$, $e_2 = (u, y)$ for `2-Opt`, and $e_1 = (x, v)$, $e_2 = (u, q)$, $e_3 = (p, y)$ or $e_1 = (x, q)$, $e_2 = (u, y)$, $e_3 = (p, v)$ for `3-Opt`; the chosen move is the one that increases the weight of the resulting matching the most.

Algorithm 1: Given a weighted bipartite graph $G = (X \cup Y, E)$ with parameters α and c, find the optimum mapping M^*.

1: Initialize a maximum weighted mapping M^* by the *Hungarian algorithm*;

2: Compute topology similarity $t(e)$ and weight $w(e)$ for each edge $e \in M^*$, where the weight $w(e) = \alpha t(e) + (1 - \alpha)s(e)$;

3: Find a candidate set S consisting of every edge $e = (x, y) \in M^*$ which satisfies the following `2-Opt` conditions (or the `3-Opt` conditions) ($\star$):

3-1. there is $e' = (u, v) \in M^*$ with $v \in prefer_Y(x)$ and $u \in prefer_X(y)$ (or there are $e' = (u, v)$, $e'' = (p, q) \in M^*$ with $v,\ q \in prefer_Y(x)$ and $u,\ p \in prefer_X(y)$);

3-2. For $e_1 = (x, v)$ and $e_2 = (u, y)$, $swap(e, e') = \{w(e_1) + w(e_2) + \alpha(t(e_1) + t(e_2))\} - \{w(e) + w(e') + \alpha(t(e) + t(e'))\} > 0$ (or $swap(e, e', e'') = \{w(e_1) + w(e_2) + w(e_3) + \alpha(t(e_1) + t(e_2) + t(e_3))\} - \{w(e) + w(e') + w(e'') + \alpha(t(e) + t(e') + t(e''))\} > 0$, for $e_1 = (x, v)$, $e_2 = (u, q)$, $e_3 = (p, y)$ or $e_1 = (x, q)$, $e_2 = (u, y)$, $e_3 = (p, v)$);

4: **while** $S \neq \emptyset$ **do**

4-1. Select an edge $e = (x, y) \in S$ with $\max\{\frac{swap(e,e')}{2}, \frac{swap(e,e',e'')}{3}\}$ where $e' = (u, v)$ and $e'' = (p, q)$;

4-2. Based on 4-1, swap e, e' with $e_1 = (x, v)$, $e_2 = (u, y)$ to obtain a new mapping $M^* \setminus \{e, e'\} \cup \{e_1, e_2\}$ and set $S = S \setminus \{e, e'\}$, or swap e, e', e'' with $e_1 = (x, v)$, $e_2 = (u, q)$, $e_3 = (p, y)$ (or $e_1 = (x, q)$, $e_2 = (u, y)$, $e_3 = (p, v)$) to obtain a new mapping $M^* \setminus \{e, e', e''\} \cup \{e_1, e_2, e_3\}$ and set $S = S \setminus \{e, e', e''\}$;

4-3. Verify if the newly inserted edges e_1, e_2 (or e_1, e_2, e_3) satisfy the above conditions ($\star$) with any other edge e_0 and put them into S if necessary;

4-4. Update the topology similarity $t(e)$ for each edge $e \in M^*$ incident to a neighbor in $N_{G_X}(x)$, $N_{G_Y}(y)$, $N_{G_X}(u)$, and $N_{G_Y}(v)$ (and $N_{G_X}(p)$, $N_{G_Y}(q)$ if necessary), and modify $w(e)$, $swap(e, e')$, and $swap(e, e', e'')$ accordingly;

end while;

5: Output the final mapping M^*;

Note that only the topology similarity $t(e)$ for each edge $e \in M^*$ incident to a vertex in $N_{G_X}(x)$, $N_{G_Y}(y)$, $N_{G_X}(u)$, $N_{G_Y}(v)$, $N_{G_X}(p)$, and $N_{G_Y}(q)$ would be changed when we swap the edges (x, y), (u, v), and (p, q) in M^*. In addition, as we consider the weight difference $w(M^* \setminus \{e, e'\} \cup \{e_1, e_2\}) - w(M^*)$ (or $w(M^* \setminus \{e, e', e''\} \cup \{e_1, e_2, e_3\}) - w(M^*)$), removing the edge $e = (x, y) \in M^*$ causes the weight loss $w(e)$, but it also causes neighbors in $N_{G_X}(x)$ and $N_{G_Y}(y)$ to lose $\alpha t(e)$. Removing the edges $e' = (u, v)$ and $e'' = (p, q)$ produces an analogous effect. On the other hand, we gain weight $w(e_1)$ as well as $\alpha t(e_1)$ from inserting the edge $e_1 = (x, v)$. Inserting the edge $e_2 = (u, y)$ (or the edges $e_2 = (u, q)$ and $e_3 = (p, y)$) produces an analogous effect. Therefore we let the weight difference, denoted $swap(e, e')$, be defined as $\{w(e_1) + w(e_2) + \alpha(t(e_1) + t(e_2))\} - \{w(e) + w(e') + \alpha(t(e) + t(e'))\}$ (and $swap(e, e', e'') = \{w(e_1) + w(e_2) + w(e_3) + \alpha(t(e_1) + t(e_2) + t(e_3))\} - \{w(e) + w(e') + w(e'') + \alpha(t(e) + t(e') + t(e''))\}$ as well). The second condition of $(\star)$ (for `2-Opt` and `3-Opt`) thus ensures that the objective function increases after the swap.

Running-time Analysis

Theorem 1 *Given a a weighted bipartite graph $G = (X \cup Y, E)$ with parameters α and c, the running time of* Algorithm 1 *is pseudo-polynomial time bounded in the worst case.*

Proof. It is readily seen that the cardinality of a maximum-weight mapping M^* is $|M^*| \leq \min\{|X|, |Y|\}$. Note that the first step of Algorithm 1 to obtain a maximum weighted mapping M^* by the *Hungarian algorithm* takes $O(|M^*|^3)$ time.

Let Δ denote the maximum degree of a vertex in G_X and G_Y, i.e. the largest number of neighbors a vertex in $X \cup Y$ can have. Let B denote the largest similarity value for two sequences, i.e. $B = \max_{x \in X, y \in Y}\{s(x, y)\}$. In Step 2, we compute the topology similarity $t(e)$ and the weight $w(e)$ for each edge $e = (x, y) \in M^*$. Since we consider all possible pairwise combinations between neighbors of x and neighbors of y, this requires $O(|M^*| \times \Delta^2)$ time.

In Step 3, we find the candidate set S. We first compute the subsets $prefer_Y(x)$ and $prefer_X(y)$ for each vertex in $X \cup Y$. The running time is bounded by $O(|X| \times |Y|)$ since it requires $O(|Y|)$ (respectively $O(|X|)$) time to find the c highest-weighted neighbors in Y (respectively X) for each vertex $x \in X$ (respectively $y \in Y$), for any constant c. We then find all the edges $e' \in M^*$ satisfying the properties $(\star)$ for every edge $e \in M^*$. For every edge $e = (x, y) \in M^*$, there are at most c^2 edges in M^* with one endpoint in $prefer_X(y)$ and the other endpoint in $prefer_Y(x)$ that e can be swapped with. Each of the c^2 possible weight differences $swap(e, e')$ and $\binom{c^2}{2}$ $swap(e, e', e'')$ can be computed in constant time from the topology similarity and sequence similarity for every edge $e \in M^*$. Hence Step 3 takes $O(c^4|M^*|)$ time.

Step 4 is an iteration, and we first consider the time complexity of one iteration. The maximum value of $swap(e, e')$ and $swap(e, e', e'')$ can be found in constant time by using a priority queue. The swap operation also takes constant time. For the two newly inserted edges of M^*, we verify if they satisfy the properties $(\star)$ in $O(c^4)$ time as above. The last step of updating the values of $t(e)$, $w(e)$, $swap(e, e')$, and $swap(e, e', e'')$ takes $O(\Delta^2)$ time, since only the edges $e \in M^*$ with one endpoint in $N_{G_X}(x) \cup N_{G_X}(u) \cup N_{G_X}(p)$ and the other, in $N_{G_Y}(y) \cup N_{G_Y}(v) \cup N_{G_Y}(q)$, are affected. Thus, each iteration requires $O(\max\{c^4, \Delta^2\})$ time.

Finally, consider the number of iterations of the while loop. The total sequence score is an integer and varies between 0 and $|M^*| \times B$, and similarly, the total topology score is an integer and varies between 0 and $|M^*|^2$; the consecutive values of $w(M^*)$ form a strictly increasing sequence whose length is bounded by $(|M^*| \times B + 1) \times (|M^*|^2 + 1) \in O(|M^*|^3 \times B)$. Since Step 4 is the dominating one, Algorithm 1 runs in $O(\max\{c^4, \Delta^2\} \times B \times |M^*|^3)$ time. We note that the k-`Opt` technique can also be pseudo-polynomial time bounded by $O(\max\{c^{2(k-1)}, \Delta^2\} \times B \times |M^*|^3)$. □

References

[1] Arya, V., Garg, N., Khandekar, R., Meyerson, A., Munagala, K. and Pandit, V. Local search heuristics for k-median and facility location problems. *SIAM J. Comput*, 33(3) (2004) pp.544–562.

[2] Chandra, B., Karloff, H. and Tovey, C. New results on the old k-opt algorithm for the TSP. In *Proceedings of the 5th ACM-SIAM Symposium on Discrete Algorithm* (1994) pp.150–159.

[3] Johnson, D.S. and McGeoch, L.A. The traveling salsman problem: a case study in local optimization. In: E.H.L. Aarts and J.K. Lenstra (eds.). Local Search in Combinatorial Optimization. John Wiley & Sons, London, (1997) pp.215–310.

[4] Kao, M.Y., Lam, T.W., Sung, W.K., and Ting, H.F. A decomposition theorem for maximum weight bipartite matchings. *SIAM J. Computing*, 31 (2001) pp.18–26.

[5] Kuhn, H.W. The Hungarian Method for the assignment problem. *Naval Research Logistics Quarterly*, 2 (1955) pp.83–97.

[6] Lueker, G. manuscript, Princeton University (1976).

[7] Papadimitriou, C.H. and Steiglitz, K. On the complexity of local search for the traveling salesman problem. *SIAM J. Computing*, 6 (1977) pp.76–83.

[8] Pardalos, P.M., Rendl, F. and Wolkowicz, H. The Quadratic Assignment Problem: A Survey and Recent Developments. In *Proceedings of the DIMACS Workshop on Quadratic Assignment Problems, volume 16 of DIMACS Series in Discrete Mathematics and Theoretical Computer Science*, (1994) pp.1–42.

[9] Taylor, R. Constrained switchings in graphs. *Combinatorial Mathematics*, 8 (1980) pp.314–336.

[10] Viger, F., Latapy, M. Efficient and simple generation of random simple connected graphs with prescribed degree sequence. In *Proceedings of the International Computing and Combinatorics Conference*, (2005) pp. 440–449.

Table S1 shows that the EC ratios improve significantly after applying the `2-Opt` and `3-Opt` heuristics to each pair of the older PPI networks; on the other hand, the FC values of the initial mappings and of those refined by PISwap do not differ substantially. This is similar to the situation observed with the more recent PPI networks in the main text.

	DM-SC			CE-SC			CE-DM			HS-MM		
	initial	`2-Opt`	`3-Opt`	initial	`2-Opt`	`3-Opt`	initial	`2-Opt`	`3-Opt`	initial	`2-Opt`	`3-Opt`
♯ of swaps	0	97	133	0	38	73	0	71	166	0	43	51
EC ratio	0.48%	0.71%	0.77%	0.67%	0.95%	1.11%	0.52%	1.01%	1.39%	29.75%	37.04%	37.66%
Functional Coherence	0.596	0.595	0.596	0.294	0.292	0.294	0.395	0.395	0.395	0.46	0.469	0.469
Running time (sec.)	236	6	28	228	4	17	1587	16	79	25263	96	887

Table S1. Evaluation of alignments based on the initial mappings produced by *Hungarian algorithm*; CE = *C. elegans*, DM = *D. melanogaster*, SC = *S. cerevisiae*, HS = *H. sapiens*, and MM = *M. musculus*

Figure S1 shows the results of using PISwap to refine the initial mappings produced by GRAAL, IsoRank, and PATH on the more recent PPI networks. Just as with the more recent PPI networks, PISwap significantly improves the EC score of these initial alignments. Similar to the results presented in the main text, the refining effects on the three alignment tools are different. The refined EC ratios are nearly double those of the initial mappings obtained by GRAAL; on the other hand, the EC ratios for IsoRank increase by 15% to 30% after refinement, and those for PATH increase dramatically by a factor of 3 to 16.

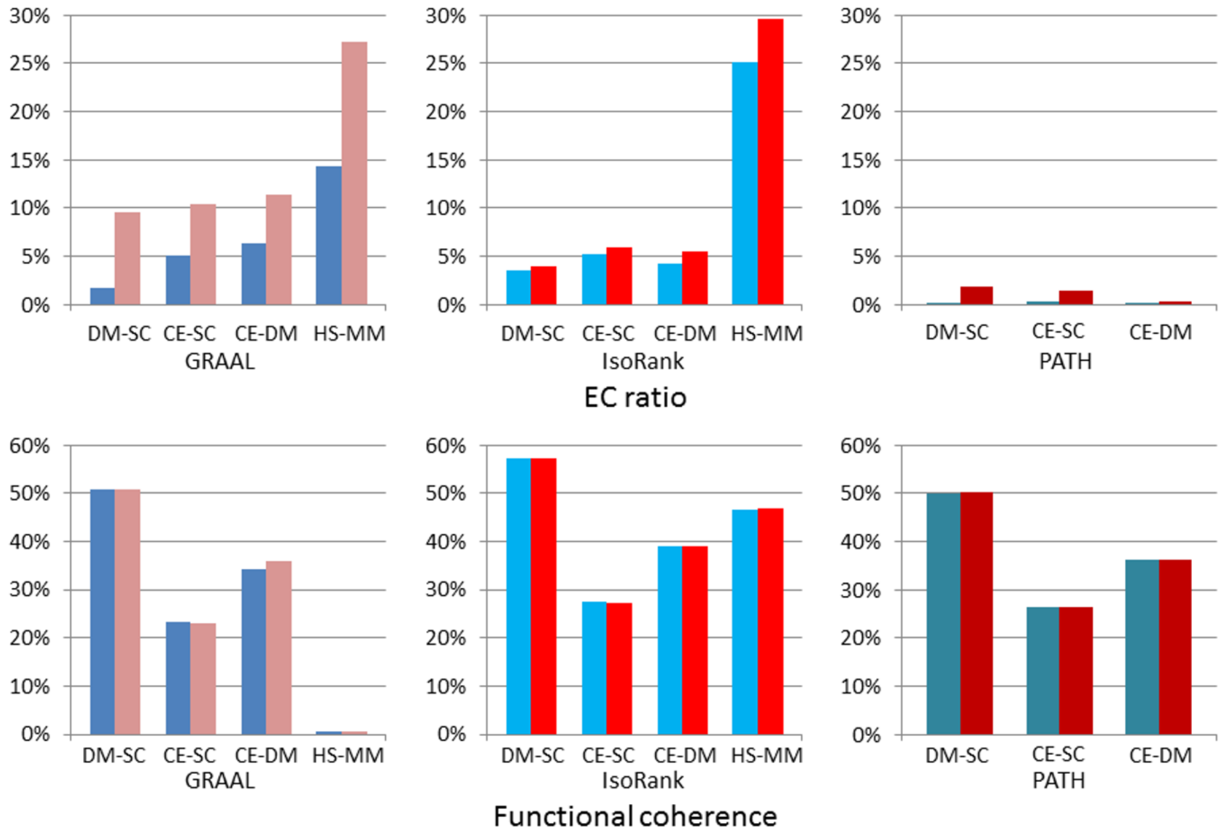

Fig. S1. Evaluation of the refinement of the initial mappings obtained by GRAAL, IsoRank and PATH; each of the blue-series and red-series bars, respectively, represents the result before and after refinement by PISwap.
